# Chemical Profile of Kumquat (*Citrus japonica* var. margarita) Essential Oil, In Vitro Digestion, and Biological Activity

**DOI:** 10.3390/foods13223545

**Published:** 2024-11-06

**Authors:** Ivana Vrca, Željana Fredotović, Blaž Jug, Marija Nazlić, Valerija Dunkić, Dina Jug, Josip Radić, Sonja Smole Možina, Ivana Restović

**Affiliations:** 1 Faculty of Science, University of Split, Ruđera Boškovića 33, 21000 Split, Croatia; ivrca@pmfst.hr (I.V.); zfredotov@pmfst.hr (Ž.F.); mnazlic@pmfst.hr (M.N.); dunkic@pmfst.hr (V.D.); 2 Biotechnical Faculty, University of Ljubljana, Jamnikarjeva ulica 101, 1000 Ljubljana, Slovenia; blaz.jug@bf.uni-lj.si (B.J.); dina.ramic12@gmail.com (D.J.); sonja.smole-mozina@bf.uni-lj.si (S.S.M.); 3 Faculty of Chemistry and Technology, University of Split, Ruđera Boškovića 35, 21000 Split, Croatia; josip.radic@ktf-split.hr; 4 Faculty of Humanities and Social Sciences, University of Split, Poljička cesta 35, 21000 Split, Croatia

**Keywords:** *Citrus japonica* var. margarita, essential oil, extraction techniques, chemical composition, in vitro digestion, biological activity

## Abstract

Kumquat is one of the smallest citrus fruits (from the Rutaceae family), and its essential oil’s biological effects have not yet been sufficiently researched, in contrast to the essential oils of its relatives. Therefore, the aim of this large-scale study was to investigate the chemical profile of kumquat essential oils (KEOs) isolated by microwave-assisted distillation (MAD) and Clevenger hydrodistillation using GC-MS analysis. To test the bioaccessibility of their bioactive components, in vitro digestion with commercially available enzymes was performed. The final step of this research was to test their cytotoxic activity against a cervical cancer cell line (HeLa), a human colon cancer cell line (HCT116), a human osteosarcoma cell line (U2OS), and a healthy cell line (RPE1). Two methods were used to test the antioxidant activity: DPPH (2,2-diphenyl-1-picrylhydrazyl) and ORAC (oxygen radical absorbance capacity). The antibacterial activity was tested in relation to the growth and adhesion of *Escherichia coli* and *Staphylococcus aureus* on a polystyrene surface. The GC-MS analysis showed that the major compound in both kumquat essential oils was limonene, which was stable before and after in vitro digestion (>90%). The results showed that the cytotoxic activity of the KEOs in all three cancer cell lines tested was IC_50_ 1–2 mg/mL, and in the healthy cell line (RPE1), the IC_50_ value was above 4 mg/mL. The antibacterial activity of the KEOs obtained after MAD and Clevenger hydrodistillation was 4 mg/mL against *E. coli* and 1 mg/mL against *S. aureus*. The KEOs after MAD and Clevenger hydrodistillation reduced the adhesion of *E. coli* by more than 1 log, while there was no statistically significant effect on the adhesion of *S. aureus* to the polystyrene surface. Both KEOs exhibited comparable levels of antioxidant activity using both methods tested, with IC50 values of 855.25 ± 26.02 μg/mL (after MAD) and 929.41 ± 101.57 μg/mL (after Clevenger hydrodistillation) for DPPH activity and 4839.09 ± 91.99 μmol TE/g of EO (after MAD) and 4928.78 ± 275.67 μmol TE/g of EO (after Clevenger hydrodistillation) for ORAC. The results obtained show possible future applications in various fields (e.g., in the food, pharmaceutical, cosmetic, and agricultural industries).

## 1. Introduction

Different citrus species are used for traditional medicinal uses, where whole fruits or peels are indicated to treat various diseases: muscle pain, skin infections, indigestion, cough, and hypertension [1,2,3,4,5,6].

Kumquat (*Citrus japonica* var. margarita) is the smallest citrus fruit that belongs to the Rutaceae family and is elliptical-shaped [1,7,8,9,10]. Cultivation of kumquat trees, which originated in Southeast China, dates back to the 12th century. Since then, it has spread to South Asia, South Africa, South and North (Florida, California) America, the Mediterranean coast, and Australia [8,11]. Kumquat essential oil (KEO) shows biological activity and can also be used as a dietary supplement in anti-obesity drugs [10,12]. Kumquat is preferred for its excellent flavor, nutrition, ability to be eaten without peeling it, and medicinal and therapeutic effects [10,12]. The citrus peel contains approximately 0.5–5% of EO among various species isolated with different extraction methods and solvents [13,14]. Due to the citrus peel being enriched with EO and flavonoids, it possesses health-promoting and biological activities [14,15]. The peel of kumquats is tasty and edible and has a characteristic aroma thanks to the presence of terpenoids and flavonoids [8,10,16]. It is also interesting to note that the flavonoid composition of kumquat is very different from that of other citrus species [7]. KEO, extracted from ripe kumquats, has significant potential value for the cosmetics, phytopharmaceutical, and food industries due to its pleasant fragrance and various biological activities [9].

The extraction of volatile and non-volatile components is the first and most significant step in research. The desired compounds can be isolated from fresh, dried or frozen plant material [17]. The most commonly used extraction method for the isolation of EO is hydrodistillation extraction (HDE), despite its shortcomings like a long duration time and low-efficiency process [18], while advanced microwave-assisted extraction techniques use electromagnetic waves that cause rapid heat transfer and destruction of the cell structure [19,20]. Additionally, microwave-assisted extraction techniques are faster, easier, and eco-friendly and allow for the extraction of bioactive components with low energy costs, quickly penetrating the medium and dissolving it volumetrically [19,20]. Therefore, the target substances can be quickly transferred from the plant matrix to the solvent [21]. EO components present in plants are basically divided into two different groups: terpenoids and phenylpropanoids [22]. In order to understand the effects of the structure and composition of food on human health, digestion in the upper gastrointestinal tract is increasingly being simulated [23]. In vitro digestion models usually comprise three phases (the oral, gastric, and intestinal phases) to determine the bioaccessibility of the desired compounds after digestion [23,24].

The health benefits of other citrus fruits are well documented, and their bioactivity is attributed to the presence of flavonoid compounds [25]. Nevertheless, compared to other citrus oils, there are few studies on the biological activity of KEO [9].

Therefore, the objectives of this research were: (1) to isolate EOs from fresh kumquat peels (*C. japonica* var. margarita) by Clevenger hydrodistillation and microwave-assisted distillation (MAD); (2) to study their chemical profile by GC-MS analysis; (3) to investigate the bioaccessibility of the bioactive components after in vitro digestion with commercially available enzymes; and (4) to test their biological activities (cytotoxic activity was tested against a cervical cancer cell line (HeLa), a human colon cancer cell line (HCT116), a human osteosarcoma cell line (U2OS), and a healthy cell line (RPE1)). In addition, (5) their antioxidant activity was also investigated using two methods: DPPH (2,2-diphenyl-1-picrylhydrazyl) and ORAC (oxygen radical absorbance capacity). (6) Their antibacterial activity against the growth and adhesion of *E. coli* and *S. aureus* on a polystyrene surface was also tested with a view to possible future application in various industries (food, pharmaceutical, cosmetics, and agro-industry).

## 2. Materials and Methods

### 2.1. Plant Material

**Figure 1:**
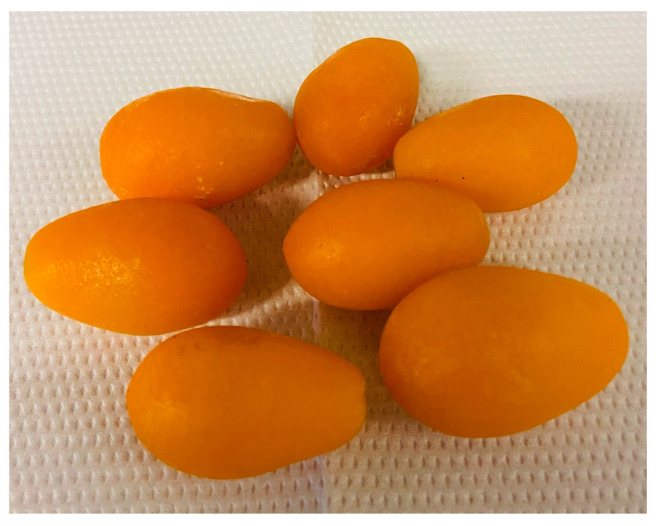
Ripe kumquat fruits from Milna on the island of Brač (Author: Ivana Vrca).

In Milna on the island of Brač, ripe kumquat fruits were harvested in March 2024 (Figure 1). The age of the plantation was 15 years, and the fruits were harvested from about 10 trees. To extract the essential oils (EOs) from the fresh kumquat fruit peels using Clevenger hydrodistillation and microwave-assisted distillation (MAD), the pulp was first separated from the peel. The kumquat peel was then crushed into smaller pieces to improve the extraction of the essential oil. Approximately 200 g of the plant material was weighed and immersed in distilled water immediately before the above extraction techniques were applied.

### 2.2. Clevenger Hydrodistillation

The EO from kumquat peel was extracted by modified Clevenger hydrodistillation for 2.5 h as described by Vrca et al. [26] with minor modifications, and the mixture of organic solvents (pentane and diethyl ether in a 2:1 ratio) was used as a trap for EO isolation. The obtained KEO was stored in vials at −20 °C until further analysis.

### 2.3. Microwave-Assisted Distillation (MAD)

The extraction of KEO was also performed by MAD using an ETHOS X microwave extraction system (Milestone, Sorisole (BG), Italy) at a power of 800 W for 30 min as described in detail by Vrca et al. [19,26] with slight modifications. The temperature in the microwave oven was about 98 °C. The KEO was also stored in vials at −20 °C until further analysis.

### 2.4. GC-MS Analysis

A gas chromatograph (model 3900; Varian Inc., Lake Forest, CA, USA) equipped with a flame ionization detector (FID), a mass spectrometer (model 2100T; Varian Inc.), a non-polar capillary column VF-5ms, and a polar capillary column CP-Wax 52 CB were used for GC–MS analysis of volatile compounds in two KEO samples. This was done in accordance with the method by Vrca et al. [27], where it is described in detail. Mass spectra was compared from the homemade library and literature [28].

### 2.5. In Vitro Digestion

The static in vitro digestion model includes a simulated digestion in three phases (oral, gastric, and intestinal) using commercial digestive enzymes with constant experimental conditions during each digestion phase according to Brodkorb et al. [23] and Minekus et al. [24]. To simulate in vitro digestion, salivary amylase, pancreatin, and bile salts were purchased from Sigma-Aldrich (St. Louis, MO, USA), Merck KgaA (Darmstadt, Germany). Rabbit gastric extract (RGE15) was purchased from Lipolytech (Marseille, France). All other chemicals and reagents were of analytical grade.

Electrolyte stock solutions (KCl, KH_2_PO_4_, NaHCO_3_, NaCl, MgCl_2_(H_2_O)_6_, (NH_4_)_2_CO_3_, HCl, CaCl_2_(H_2_O)_2_) were prepared at stock concentrations and used in the preparation of digestive fluids (simulated saliva, simulated gastric and intestinal fluids) according to INFOGEST 2.0 [23]. The oral phase comprised the dilution of the KEOs after Clevenger hydrodistillation and MAD in a ratio of 1:1 (*v*/*v*) with a simulated salivary fluid containing salivary amylase (15 U/mL) for 2 min, temperature of 37 °C, and pH 7. The final volume of the oral bolus was 4 mL. The oral bolus was then diluted 1:1 with 4 mL of simulated gastric fluid (SGF) containing pepsin and gastric lipase (2000 U/mL, 60 U/mL) and incubated with agitation at a pH of 2 for 1 h at a temperature of 37 °C. At the end of in vitro digestion, the gastric mucus was diluted 1:1 with simulated intestinal fluid (SIF). Pancreatin and bile salts were added to the SIF and incubated for 2 h at a temperature of 37 °C and a pH value of 7.0 (trypsin activity 100 U/mL, bile salts 10 mM). The final concentration of KEO in the samples after digestion was 2 mg/mL in a final volume of 16 mL. After incubation, all samples were briefly placed on ice (5 min) to stop the enzymatic reaction. The digested samples were then centrifuged using the Hettich Universal 32 R centrifuge D-78532 (Tuttlingen, Germany) for 5 min at 4000 rpm at room temperature. After centrifugation, the supernatant and pellet of all samples were extracted with a mixture of pentane : diethyl ether = 3:1. All digestion experiments were performed in duplicate (n = 3). The bioaccessibility of the main compounds in KEOs after Clevenger hydrodistillation and MAD techniques represents the ratio of their concentrations in the supernatants (digested samples after digestion) and the concentrations before in vitro digestion. 

### 2.6. Cytotoxic Activity

The cytotoxic activity of KEOs obtained using Clevenger hydrodistillation and MAD extraction techniques was examined in vitro on cancer cell lines: human cervical cancer (HeLa), colon cancer (HCT116), human osteosarcoma (U2OS) cell line, and healthy human retinal pigmented epithelial cells (RPE1). The method was performed with MTS-based CellTiter 96^®^ Aqueous Assay (Promega, Madison, WI, USA) as described in detail by Fredotović et al. [29]. The cells were allowed to grow in an incubator at 37 °C and 5% CO_2_ until they had reached the confluence required for the experiment (80%). They were counted with an automated handheld cell counter (Merck, Darmstadt, Germany). After counting, cells were seeded in 96-well plates with serial dilutions of KEOs and allowed to grow for 48 h. At the end of the treatment, 20 µL of MTS tetrazolium reagent (Promega, Madison, WI, USA) was added to each well, and the plate was left in the incubator for 3 h. The absorbance was then measured at 490 nm using a 96-well plate reader (Infinite M Plex, Tecan, AG, Männedorf, Switzerland).

### 2.7. Bacterial Strains and Growth Conditions

For antibacterial testing, Gram-negative *E. coli* ATCC 11229 and Gram-positive *S. aureus* ATCC 25923 (Laboratory for Food Microbiology, Biotechnical Faculty, University of Ljubljana) were used. The growth conditions for the above-mentioned bacteria are described in detail by Vrca et al. [30].

### 2.8. Antibacterial Susceptibility

To determine the minimal inhibitory concentration (MIC), the KEOs were dissolved in absolute ethanol at a concentration of 10 mg/mL and diluted following Clevenger hydrodistillation and MAD techniques, as explained in detail by Klančnik et al. [31]. The lower conversion of INT to red formazan indicates that the MIC values are the lowest concentration of KEOs that significantly inhibit bacterial growth. The ethanol content in the test was never higher than 1% to avoid any effect on the development of the selected bacteria. The lowest concentration at which the bacteria did not grow was determined as MBC. 

### 2.9. Bacterial Growth Kinetics

Kumquat essential oils (KEOs) after Clevenger hydrodistillation and MAD were added to 5 mL of *E. coli* and *S. aureus* cultures to obtain final concentrations of the preparations from 4 mg/mL to 0.0625 mg/mL according to method described by Vrca et al. [30,32] with minor modifications. Cultures (*E. coli* or *S. aureus*) without the addition of KEOs were used as positive controls. MH broth with or without the addition of KEOs at different concentrations was used as a negative control and subtracted from the results obtained after the measurements. A Varioskan LUX reader (Thermo Fisher Scientific, Waltham, MA, USA) was used to measure absorbance at 600 nm every 30 min for 24 h at 37 °C to obtain growth curves.

### 2.10. Antiadhesion Assay

The adhesion of *E. coli* and *S. aureus* was analyzed after treatment with KEOs obtained after Clevenger hydrodistillation and MAD techniques. Inocula were prepared as described above and treated with KEOs at MIC, ½ MIC, ¼ MIC, and ⅛ MIC concentrations. CFU/mL was used to measure the adhesion of the cells, as previously described by Šikić Pogačar et al. [33]. An untreated culture was used as a negative control. The experiments were performed in triplicate as three or more independent experiments.

### 2.11. Antioxidant Activity

#### 2.11.1. Measurement of the ORAC Values

Using 96-well black polystyrene microtiter plates (Porvair Sciences, Leatherhead, UK), the assay was carried out in a Tecan Infinite 200 PRO spectrophotometer (Tecan Trading AG, Männedorf, Switzerland) according to a method outlined by Fredotovic et al. [29], with some modifications because of various extracts. Each plate was prepared as follows: a blank (phosphate buffer) was created in the first column, followed by an EO concentration of 50 µg/mL in the second, and 25 µg/mL in the third. After adding 30 µL of blank or extract to each well for each of the above columns, 180 µL of fluorescein (1 µM) and 70 µL of 2,2′-azobis(2-methyl-propionamidine) dihydrochloride (AAPH, Acros Organics, Waltham, MA, USA) (300 mM) were added to start the reaction. A calibration curve was prepared after a separate reaction plate for the reference standard Trolox (6.25–50 µM) (Sigma-Aldrich) was completed. After the reaction occurred in a phosphate buffer (0.075 mM, pH 7.0), both KEOs were diluted in acetone to a concentration of 10 mg/mL. They were then diluted to the test concentrations of 50 µg/mL and 25 µg/mL. µmol Trolox equivalents (TE) per milligram KEO was the unit of measurement for the ORAC values of the KEOs. The results were determined in three separate experiments. 

#### 2.11.2. Measurement of the DPPH Radical Scavenging Activity

The antioxidant capacity of the extracts utilized in this work was assessed using the DPPH method, which was previously reported by Mensor et al. [34] and Payet et al. [35] and modified for the tested plant extracts. This technique was also used with clear polystyrene 96-well microtiter plates (Porvair Sciences, Leather-head, UK) in a Tecan Infinite 200 PRO spectrophotometer. KEOs were used for the assay as specified in the ORAC technique; however, they were diluted to 1 mg/mL for this reaction. The further method was done as previously described in [36]. Each measurement was carried out three times. To facilitate comparison with data from other relevant publications, the results were expressed as IC50 values in µg/mL EO solution. 

### 2.12. Statistical Analysis

Statistical analysis of in vitro digestion, cytotoxic, antioxidant, and antiadhesion activities was performed using GraphPad Prism Version 9 (GraphPad Software, Inc., Boston, MA, USA).

A two-way ANOVA test was used for the statistical analysis of the data. Then, to examine the difference between main compounds in KEOs before and after in vitro digestion (different letter a–b), Tukey’s multiple comparison test with a significance level of *p* < 0.05 was used. The results are presented as mean ± SD (n = 3).

Cytotoxic activity of KEOs (IC_50_ values) is given as mean values of three independent experiments performed in groups of four ± SD. Statistical analysis was performed using two-way ANOVA followed by a Sidak’s multiple comparisons test. IC_50_ values were calculated from three independent experiments.

For antiadhesion activity, IBM SPSS Statistics 23 (Statsoft Inc., Tulsa, OK, USA) was also used to perform statistical analyses. The Kolmogorov–Smirnov test of normality was used to determine the distribution of the data and to determine *p*-value < 0.05 with *t*-tests for two independent means.

## 3. Results

### 3.1. GC-MS Analysis of Kumquat Essential Oil Before and After In Vitro Digestion

The importance of this research lies in the study of the differences in the chemical profiles of the under-researched kumquat essential oils (KEOs) obtained by two extraction techniques: the conventional extraction technique of Clevenger hydrodistillation and modern extraction technique of microwave-assisted distillation (MAD). Considering the almost identical chemical compositions of KEOs obtained by both extraction techniques, the MAD technique is the better choice due to its advantages such as shorter duration, environmentally friendly chemistry, lower power consumption, and ease of use. The most abundant compound in the KEOs was limonene, while *β*-pinene and germacrene D were the least important components (Table 1, Figures S3–S6). Since the whole kumquat fruit is eaten together with the peel, the amount of orally ingested KEOs is very interesting and important. Therefore, digestion of KEOs was also performed to determine the bioaccessibility of bioactive components after in vitro digestion with commercially available enzymes. The results showed that *β*-pinene and limonene (the major components of KEOs) were stable after in vitro digestion. The minor compound germacrene D was considerably degraded after digestion (about 20% for both KEOs) but was still stable to a large percentage.

From the results obtained after in vitro digestion, it can be concluded that the main components of KEOs are stable and bioaccessible. Therefore, the continuation of the research included testing the biological activity (cytotoxic, antioxidant, antibacterial, and antiadhesive activities).

### 3.2. Cytotoxic Activity of Isolated KEO After Clevenger Hydrodistillation and Microwave-Assisted Distillation (MAD)

**Figure 2:**
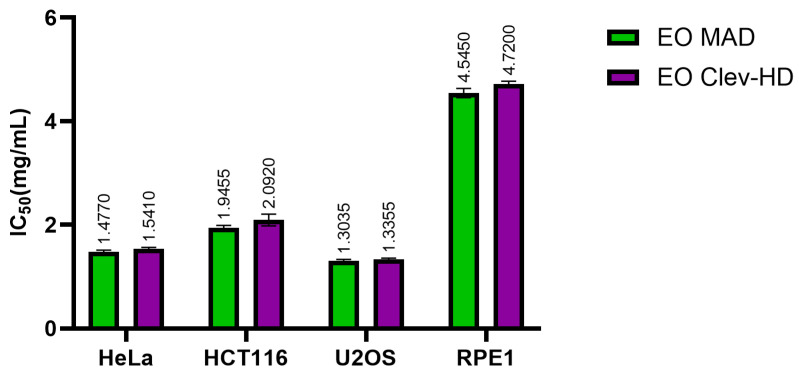
Cytotoxic activity of kumquat essential oils obtained after microwave-assisted distillation (MAD) (EO MAD) and Clevenger hydrodistillation (EO Clev-HD) on HeLa, HCT116, and U2OS cancer cell lines and RPE1 cell line. IC_50_ values are given as mean values of three independent experiments performed in groups of four ± SD (standard deviation). Statistical analysis was performed using two-way ANOVA followed by Sidak’s multiple comparisons test. There was no significant difference (*p* > 0.05).

The cytotoxic activity of KEOs was investigated in cervical (HeLa), colon (HCT116), and osteosarcoma (U2OS) cell lines as well as in a healthy cell line (RPE1). The results showed that both KEOs exhibited moderate activity against the cancer cell lines and remarkable resistance against the healthy cell line, retinal pigmented epithelial cells (RPE1) (Figure 2). The KEOs (EO MAD) obtained after microwave-assisted distillation (MAD) showed the best cytotoxic activity with an IC_50_ value of 1.3035 mg/mL against the U2OS cancer cell line. Against the Hela and HCT116 cancer cell lines, the IC50 was 1.4770 mg/mL and 1.9945 mg/mL, respectively. Similar results were obtained for the KEOs obtained after Clevenger hydrodistillation (Figure 2).

### 3.3. Antioxidant Activity

The antioxidant activity of KEOs was tested using two methods: ORAC (oxygen radical absorbance capacity) and DPPH (2,2-diphenyl-1-picrylhydrazyl). KEOs after MAD and Clevenger hydrodistillation were tested.

#### 3.3.1. ORAC Activity

Table 2 shows the antioxidant activity of KEOs according to MAD and Clevenger hydrodistillation. There were no significant differences between antioxidant activities from EOs extracted with two methods.

#### 3.3.2. DPPH Activity

In the DPPH assay (Table 2), there was also no significant difference between the activities of the two extracts. These results are consistent with other activities of these EOs and also with a chemical composition that was similar in both extraction methods.

### 3.4. Antibacterial Activity

Table 3 presents the antibacterial activity of KEOs after Clevenger hydrodistillation and MAD against Gram-negative *E. coli* and Gram-positive *S. aureus*. The results indicate that the MICs of KEOs were 4 mg/mL against *E. coli* and 1 mg/mL against *S. aureus* for both extraction methods. However, bactericidal concentrations were not obtained for either the bacterium or extraction method.

### 3.5. Bacterial Growth Kinetics

**Figure 3:**
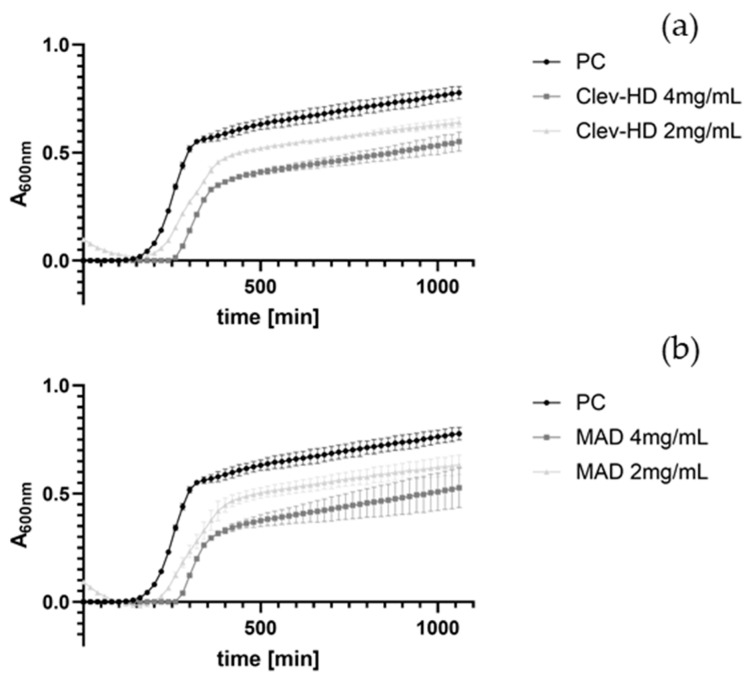
Effects of KEOs at different concentrations (mg/mL) on the growth of *E. coli* after Clev-HD (**a**) and MAD (**b**). Only statistically significant concentrations, along with the first non-significant concentration, are shown alongside the positive control (PC). Cultures were aerobically incubated for 24 h at 37 °C. Negative controls were deducted from the obtained results. Average values of A_600nm_ ± SD are shown.

**Figure 4:**
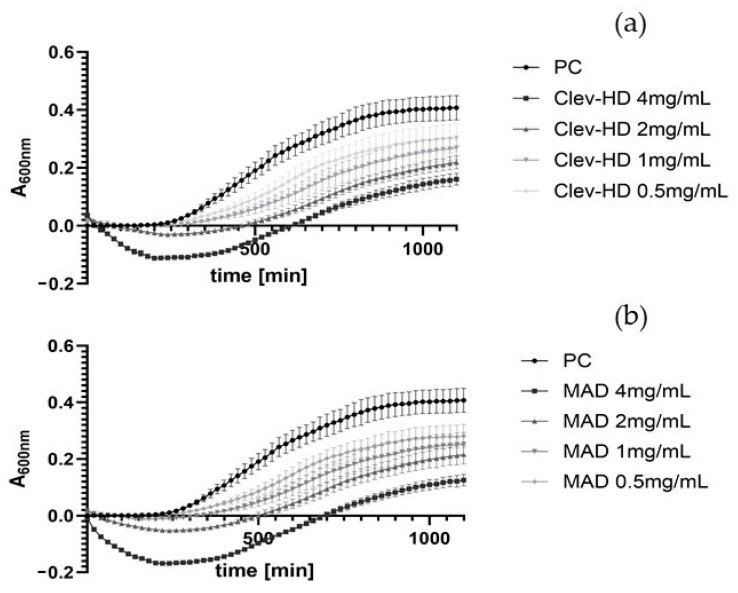
Effects of KEOs in different concentrations (mg/mL) on the growth of *S. aureus* after Clev-HD (**a**) and MAD (**b**). Only statistically significant concentrations, along with the first non-significant concentration, are shown alongside the positive control (PC). Cultures were aerobically incubated for 24 h at 37 °C. Negative controls were deducted from the obtained results. Average values of A_600nm_ ± SD are shown.

KEOs extracted after Clevenger hydrodistillation and the MAD techniques were tested for their effects on the growth of *E. coli* and *S. aureus* at concentrations ranging from 0.0625 to 4 mg/mL over 24 h (Figure 3, Figure 4, Figures S1 and S2). Both EO samples prolonged the lag phase of *E. coli* only at a concentration of 4 mg/mL. Specifically, MAD (Figure 3b) prolonged the lag phase by 170 min (from 100 to 270 min), while Clevenger prolonged it by 150 min (Figure 3a). Even more pronounced effects were observed against *S. aureus*, with MAD (Figure 4b) prolonging the lag phase from 120 min to 340, 300, and 260 min at concentrations of 4, 2, and 1 mg/mL, respectively. Similarly, KEO from Clevenger hydrodistillation (Figure 4a) prolonged the lag phase to 360, 300, and 180 min at the same concentrations (Figure 4). Based on these results, the MIC was set at 4 mg/mL for *E. coli* (Figure 3) and 1 mg/mL for *S. aureus* (Figure 4).

### 3.6. Antiadhesion Activity

**Figure 5:**
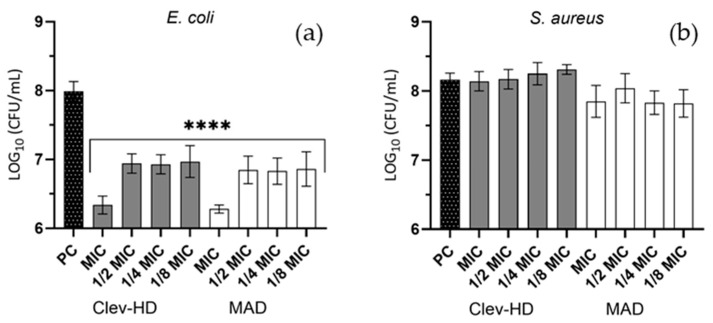
Effects of KEOs obtained after Clev-HD (gray color) and MAD (white color) in concentrations of MIC, ½, ¼, and ⅛ MIC on the adhesion to polystyrene surface of *E. coli* (**a**) and *S. aureus* (**b**). The results are expressed as means ± SD, **** *p*-value < 0.0001.

Kumquat essential oils (KEOs) extracted from fresh kumquat peel after Clevenger hydrodistillation and MAD techniques were evaluated for their activity against the adhesion of *E. coli* and *S. aureus* to the polystyrene surface. An effect on adhesion reduction was observed, with KEOs after MAD and Clevenger hydrodistillation reducing the adhesion of *E. coli* by more than 1 log (*p* < 0.0001) (Figure 5a), while there was no statistically significant effect on *S. aureus* (Figure 5b).

## 4. Discussion

The chemical profile, in vitro digestion with commercial enyzmes, and biological activity of kumquat essential oils (KEOs) obtained by advanced microwave-assisted distillation (MAD) and conventional Clevenger hydrodistillation were studied. Two KEOs were obtained and analyzed by GC-MS before and after in vitro digestion; the data obtained are shown in Table 1. This research also included cytotoxic activity of KEOs against cervical (HeLa), human colon (HCT116), and human osteosarcoma (U2OS) cancer cell lines and against a healthy cell line (RPE1). The antioxidant activity of KEOs was examined with two methods: DPPH and ORAC. The antibacterial activity of KEOs was determined against growth and adhesion to the polystyrene surface of *E. coli* and *S. aureus*.

GC-MS analysis showed that the components in the highest percentages in KEOs were limonene, *β*-pinene, and germacrene D. According to Koyasako et al. [37], after simultaneous distillation and extraction, in KEOs, the major compound was limonene with 93%, which is following our obtained results. Also, some other minor components that were not found in these EOs were identified by Koyasako et al. [37]. According to Yu et al. [38], Peng et al. [39], Umano et al. [40], and Choi [41], limonene was also a predominant compound in KEOs, while Quijano and Pino [42] reported that limonene was the most abundant compound, comprising 76.7% of peel oil using the hydrodistillation technique for 3 h. In addition to limonene, myrcene, germacrene D, and linalool were found as main components in KEOs [42]. According to Ruiz and Flotats [43], limonene was the dominant compound in various citrus EOs, which is also in accordance with the obtained results. Additionally, prior studies have demonstrated the anti-inflammatory, anti-cancer, antioxidant, antidiabetic, antiviral, and gastroprotective properties of limonene [44,45].

It is becoming increasingly necessary to simulate the digestion of the upper gastrointestinal tract to know how the composition and structure of food affect human health [23]. For this improved in vitro digestion method, the use of the rabbit gastric extract (RGE) is recommended due to gastric lipase [23]. The bioaccessibility rate can be presented by in vitro/ex vivo digestion methods, which mostly consist of two phases—gastric and intestinal. Characteristics such as sample properties, enzymatic activity, ion composition, duration time of digestion, and pH levels have important roles on the results obtained by in vitro digestion experiments [46]. Obtained results after in vitro digestion of KEOs showed that the main compound limonene was stable and bioaccessible after in vitro digestion in both KEO samples. Although research on the in vitro digestion of essential oils or extracts containing volatile compounds is rare, Vrca et al. [30] reported that the stability of the pure compound aliphatic isothiocyanate (allyl isothiocyanate) after digestion (especially the intestinal phase) with commercial enzymes is significantly lower. Vrca et al. [26] also reported in vitro and ex vivo digestion of *Tropaeolum majus* L. EO and the pure compound benzyl isothiocyanate. The results showed that after in vitro digestion, the stability of both samples was high (>70%).

The cytotoxic activity of KEOs was examined on three cancer cell lines, HeLa, HCT116, and U2OS, and one healthy cell line, RPE1. Baik et al. [47] reported that the majority of the tested citrus EOs showed no cytotoxicity in a human dermal fibroblast cell line using MTT assay, which enables their future applications in cosmetic products. In the study by Fitsiou et al. [48], KEOs were shown not to affect the viability of HepG2 and MCF-7 cells, while Caco2 and THP-1 cells showed similar sensitivity to the effects of the oils. The n-hexane extract of kumquat at an extremely high concentration of 100 mg/mL showed significant cytotoxic activity on prostate cancer cells (LNCaP). The authors hypothesized that this activity is due to the presence of β-carotene, β-cubebene, and hexadecanoic acid or is a consequence of the synergy of all biologically active substances contained in the extract [15].

The antioxidant potential of citrus EOs as free radical scavengers may lie in the antioxidant activity of limonene, the main compound of citrus oils [49]. Antioxidant activity was found to be similar for both tested extraction methods for ORAC and DPPH activity. There is not much reported data for antioxidant activities of KEOs. Nouri and Ali Shafaghatlonbar [16] found that the DPPH activity of kumquat peel essential oils was 112 µg/mL, which was a slightly higher activity than ours and which may have resulted from DPPH activity different methods use. Other citrus EOs have been reported to have higher antioxidant activity than KEOs [50]. Boudries et al. [51] reported that mandarin oils showed the strongest activity compared to clementine and wilking EOs. Studies also showed that d-limonene and *Citrus karna* EOs have a similar antioxidant potential (39.6% and 38.3%, respectively) [52]. According to Frassineti et al. [53], oils from mandarin, lemon, sweet orange, and bitter orange showed 20–70% DPPH inhibition in free radical scavenging activity. Lemon oil demonstrated the highest antioxidant capacity, with a 70% DPPH inhibition rate [53].

Citrus EOs that were studied demonstrated the antioxidant ability of scavenging DPPH and ABTS free radicals in a dose-dependent manner, according to Lin et al. [54]. With IC50 values of 15.20 mg/mL for the DPPH assay and 0.80 mg/mL for the ABTS assay, Nanfeng mandarin EOs demonstrated the highest level of antioxidant activity [54].

Terpenes and terpenoids have antimicrobial activity mainly due to their interaction with cell surface structures [55,56,57]. Gram-negative bacteria like *E. coli* are generally more resistant to higher concentrations of EOs compared to Gram-positive bacteria such as *S. aureus* due to the protective outer membrane surrounding their cell wall [58,59,60]. This observation is consistent with the results obtained in this study (Table 3). It was shown that rosemary, tea tree, thyme, and cedarwood oils, similarly to our findings, have moderate antibacterial activity against *S. aureus* [58]. D’Arrigo et al. further demonstrated that tea tree oil can enhance the efficacy of antibiotics, lowering the MIC values of tobramycin from 0.39 mg/L to even lower levels for *E. coli* and from 0.20 mg/L for *S. aureus* [61]. Interestingly, when the EOs were tested for their effects on the adhesion of *E. coli* and *S. aureus* to polystyrene surfaces, they demonstrated a stronger inhibitory effect on the Gram-negative *E. coli*. This suggests that even though bacterial growth was not significantly affected at lower concentrations, the ability of *E. coli* to adhere was significantly impaired. These findings highlight the versatility of KEOs and their broad-spectrum antibacterial properties. Furthermore, in the study of Abers et al. [58], it was shown that EOs’ antimicrobial activity in the airborne evaporative state is very effective. The best broad-spectrum antibacterial agents were shown to be rosemary, tea tree, and cassia [58].

When compared to the EOs of *Tropaeolum majus* L., as shown by Vrca et al. [30], *T. majus* EOs displayed superior antimicrobial properties, with lower MIC values and more pronounced anti-adhesion effects against the same bacterial strains. This indicates that *T. majus* EOs could be more effective as an antimicrobial agent. Essential oils from Fortunella crassifolia Swingle peel showed no selectivity in antimicrobial activity based on the cell wall differences of Gram-negative and Gram-positive bacteria, even though contrary to our results, MIC values of the EOs for *E. coli* and S. aureus were much lower (0.05 and 0.525 mg/mL) [62]. The limonene predominance in the EOs extracted from the peel of these kumquat species may be the reason for their comparatively low antibacterial activity. It has been shown that limonene has weak antibacterial properties since it is extremely volatile and hydrophobic [54,63]. These findings highlight the diverse bioactivities of essential oils, which can vary significantly depending on the type of oil, the extraction method, and the specific biological target. Overall, the results suggest that KEOs hold promise as natural agents for use in cosmetics, medicine, pharmaceuticals, and the food industry in the near future [62,63,64,65]

## 5. Conclusions

Kumquat is one of the smallest fruits belonging to the citrus family (Rutaceae), and its essential oil also exhibits various biological activities. The results showed that the major compounds in kumquat essential oils (KEOs) after Clevenger hydrodistillation and MAD techniques are limonene, *β*-pinene, and germacrene D. All compounds mentioned were stable/bioaccessible after in vitro digestion with commercially available enzymes. The cytotoxic activity of KEOs in all three cancer cell lines tested was IC_50_ 1–2 mg/mL, and in the healthy cell line (RPE1), IC_50_ was above 4 mg/mL. Both KEO samples effectively prolonged the lag phase of *E. coli* at a concentration of 4 mg/mL and of *S. aureus* at concentrations of 4, 2, and 1 mg/mL. Although the adhesion of *S. aureus* remained unchanged, a significant reduction in *E. coli* adhesion was observed, indicating strong potential for biofilm prevention. Both KEOs displayed similar antioxidant activity. The results show that KEOs can be used in various industries such as food, pharmaceuticals, cosmetics, and agriculture.

## Figures and Tables

**Table 1:** Chemical composition of kumquat essential oils (KEOs) after Clevenger hydrodistillation and microwave-assisted distillation (MAD).

Bioactive Compounds in KEO	RI	RT	Chemical Composition of EO_Clev-HD_ (%) Before In Vitro Digestion	Chemical Composition of EO_Clev-HD_ (%) After In Vitro Digestion	Chemical Composition of EO_MAD_ (%) Before In Vitro Digestion	Chemical Composition of EO_MAD_ (%) After In Vitro Digestion
*β*-pinene	979	9.41	1.52 ± 0.02 ^a^	1.42 ± 0.04 ^a^	1.38 ± 0.00 ^a^	1.37 ± 0.00 ^a^
Limonene	1030	11.14	94.91 ± 0.38 ^a^	93.77 ± 0.34 ^b^	94.18 ± 0.13 ^a^	93.30 ± 0.08 ^b^
Germacrene D	1481	30.96	1.68 ± 0.02 ^a^	1.35 ± 0.04 ^a^	1.74 ± 0.09 ^a^	1.42 ± 0.01 ^a^
Total (%)	/	/	98.11 ± 0.02 ^a^	96.54 ± 0.26 ^b^	97.29 ± 0.22 ^a^	96.09 ± 0.09 ^b^
Bioacc. (%)	/	/	/	98.40	/	98.77

Using the VF5-ms identification approach, retention indices (RIs) were calculated in relation to a range of n-alkanes (C8–C40) on capillary columns: SD represents the standard deviation of the triplicate analysis (n = 3); RI represents the comparison of RIs with those in a self-generated library published in the literature [28] and/or with real samples; and mass spectra for comparison with those in the NIST02 and Wiley 9 mass spectral libraries. Bioacc.—Bioaccessibility. After statistical data processing using a two-way ANOVA test, the main chemicals in KEOs before and after in vitro digestion were compared using Tukey’s multiple comparison test (different letters a and b), with *p* < 0.05. The results (n = 3) are given as mean ± standard deviation (SD).

**Table 2:** ORAC and DPPH antioxidant activity of two kumquat essential oils (KEOs).

Extraction/Method	Clev-HD	MAD	Trolox
ORAC	4928.78 ± 275.67 ^a^	4839.09 ± 91.99 ^a^	-
DPPH	929.41 ± 101.57 ^b^	855.25 ± 26.02 ^b^	38.94 ± 0.19 ^a^

ORAC, oxygen radical absorbance capacity, results are expressed as µmol of Trolox equivalents (TE) per g of essential oil; DPPH, results for Trolox and KEO expressed as IC_50_ in µg/mL; SD = standard deviation of triplicate analysis. For the DPPH activity, a two-way ANOVA test was used for statistical data processing, after which Tukey’s multiple comparison test was used to examine the difference between antioxidant activity of extracts and standard Trolox (different letters a and b), *p* < 0.05. For the ORAC activity, significant differences were determined using a *t*-test. ^a,b^—Mean values in the same row with different superscript letters indicate a statistically significant difference between data from two extracts (*p* < 0.05).

**Table 3:** Minimal inhibitory concentration (MIC; mg/mL) and minimal bactericidal concentration (MBC; mg/mL) against *E. coli* ATCC 11229 and *S. aureus* ATCC 25923 for kumquat essential oils after microwave-assisted distillation (MAD) and Clevenger hydrodistillation.

Kumquat Essential Oil (KEO) Samples	*E. coli* ATCC 11229		*S. aureus* ATCC 25923	
	MIC (mg/mL)	MBC (mg/mL)	MIC (mg/mL)	MBC (mg/mL)
KEO after Clev-HD	4	<4	1	<4
KEO after MAD	4	<4	1	<4

Clev-HD—Clevenger hydrodistillation; MAD—microwave-assisted distillation.

## Data Availability

The original contributions presented in the study are included in the article/Supplementary Materials, further inquiries can be directed to the corresponding author.

## Supplementary Materials

The following supporting information can be downloaded at: https://www.mdpi.com/article/10.3390/foods13223545/s1, Figure S1. Effects of KEOs at different concentrations (mg/mL) on the growth of *E. coli* after Clev-HD (a) and MAD (b); Figure S2. Effects of KEOs in different concentrations (mg/mL) on the growth of *S. aureus* after Clev-HD (a) and MAD (b); Figure S3. Chromatogram of chemical composition of EO _Clev-HD_ before in vitro digestion; Figure S4. Chromatogram of chemical composition of EO _Clev-HD_ after in vitro digestion; Figure S5. Chromatogram of chemical composition of EO _MAD_ before in vitro digestion; Figure S6. Chromatogram of chemical composition of EO _MAD_ after in vitro digestion.
