# Supplementary material for: Chemical Profile of Kumquat (Citrus japonica var. margarita) Essential Oil, In Vitro Digestion, and Biological Activity
Source: Foods. 2024 Nov 6;13(22):3545. doi: 10.3390/foods13223545 (PMC11594046; doi:10.3390/foods13223545)
Supplement: Supplementary file 1 [file foods-13-03545-s001.zip › foods-3281313-supplementary.pdf]

a)

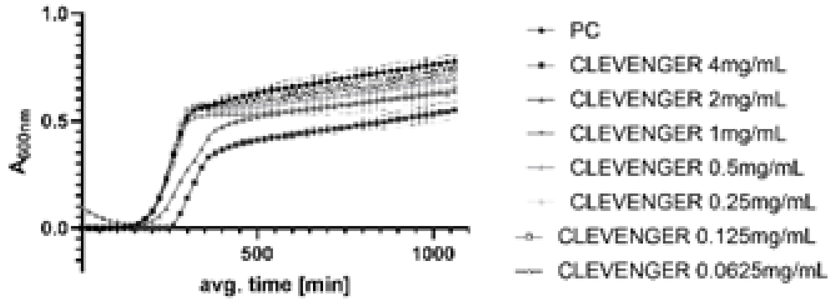

b)

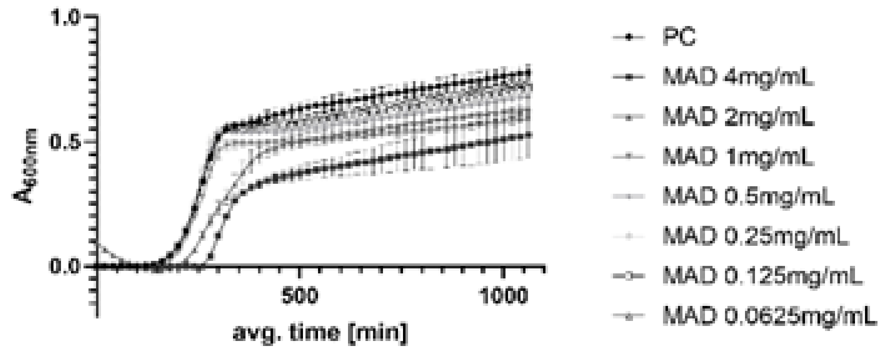

Figure S1. Effects of KEOs at different concentrations (mg/mL) on the growth of *E. coli* after Clev-HD (a) and MAD (b).

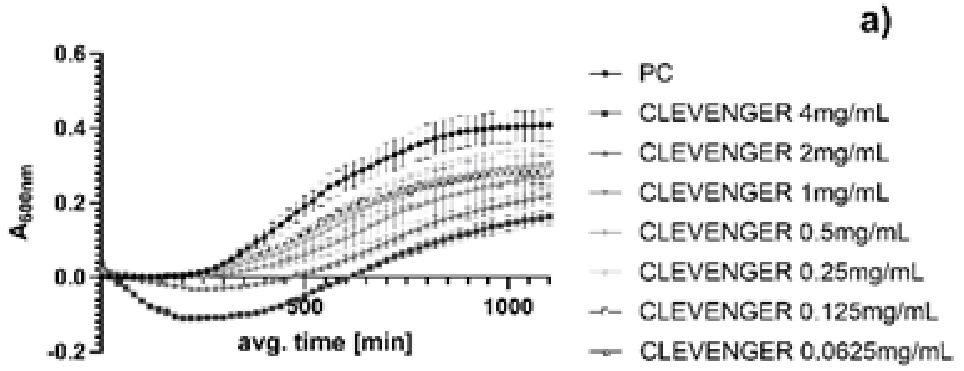

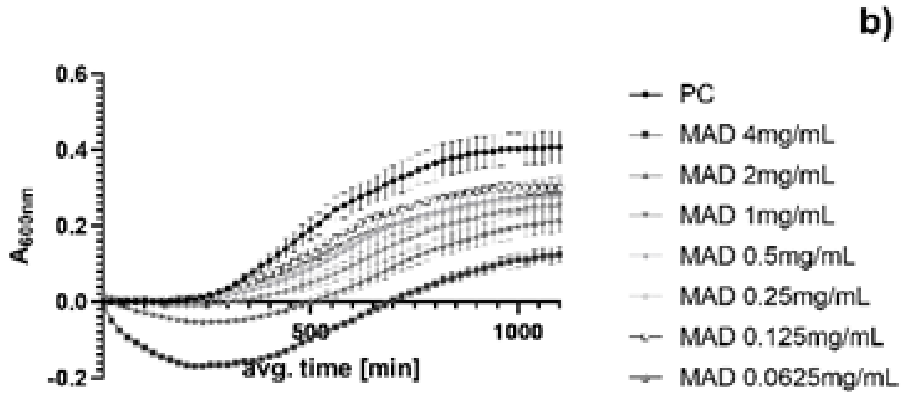

Figure S2. Effects of KEOs in different concentrations (mg/mL) on the growth of *S. aureus* after Clev-HD (a) and MAD (b).

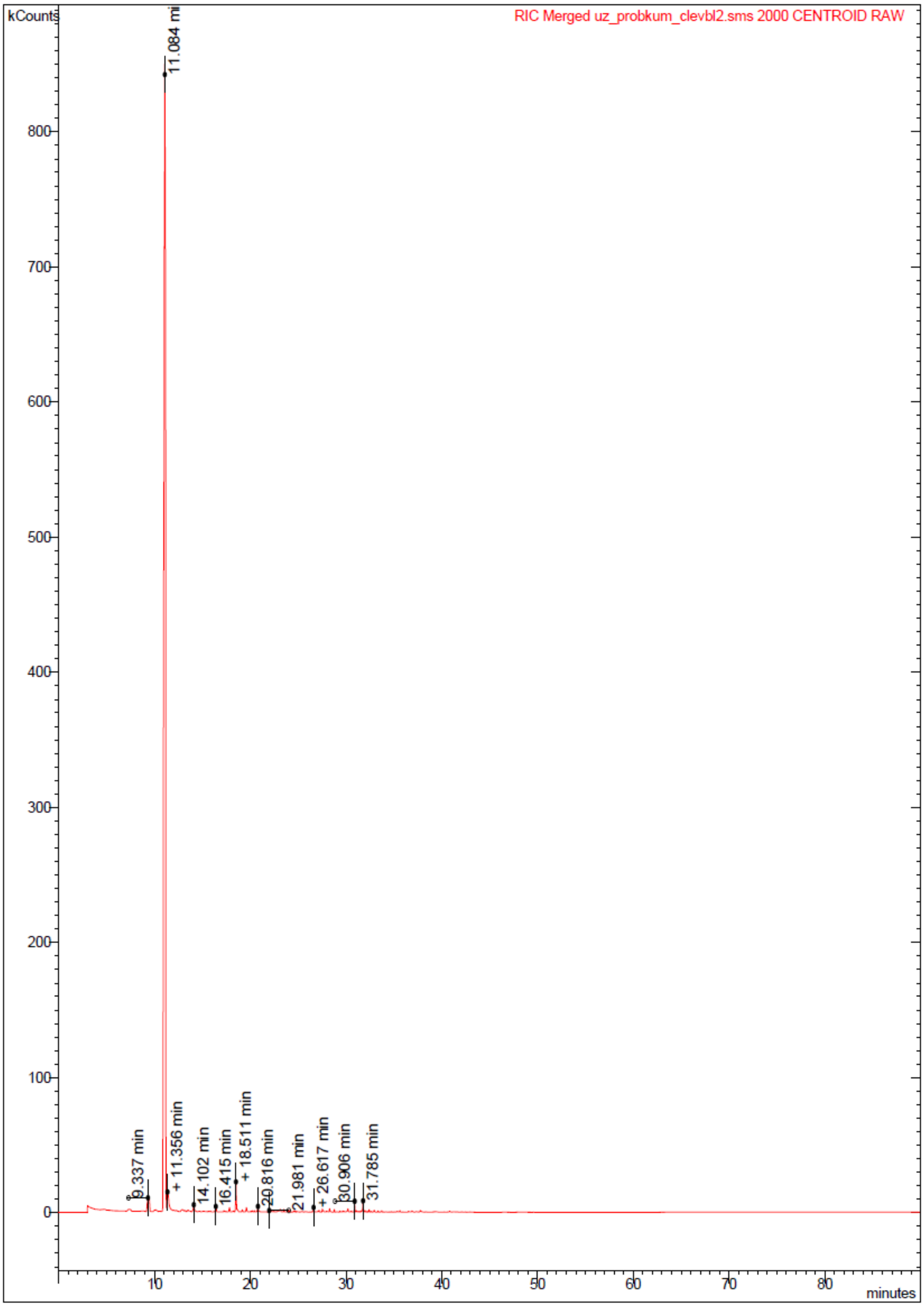

Figure S3. Chromatogram of chemical composition of EO $_{\text{Clev-HD}}$ before *in vitro* digestion.

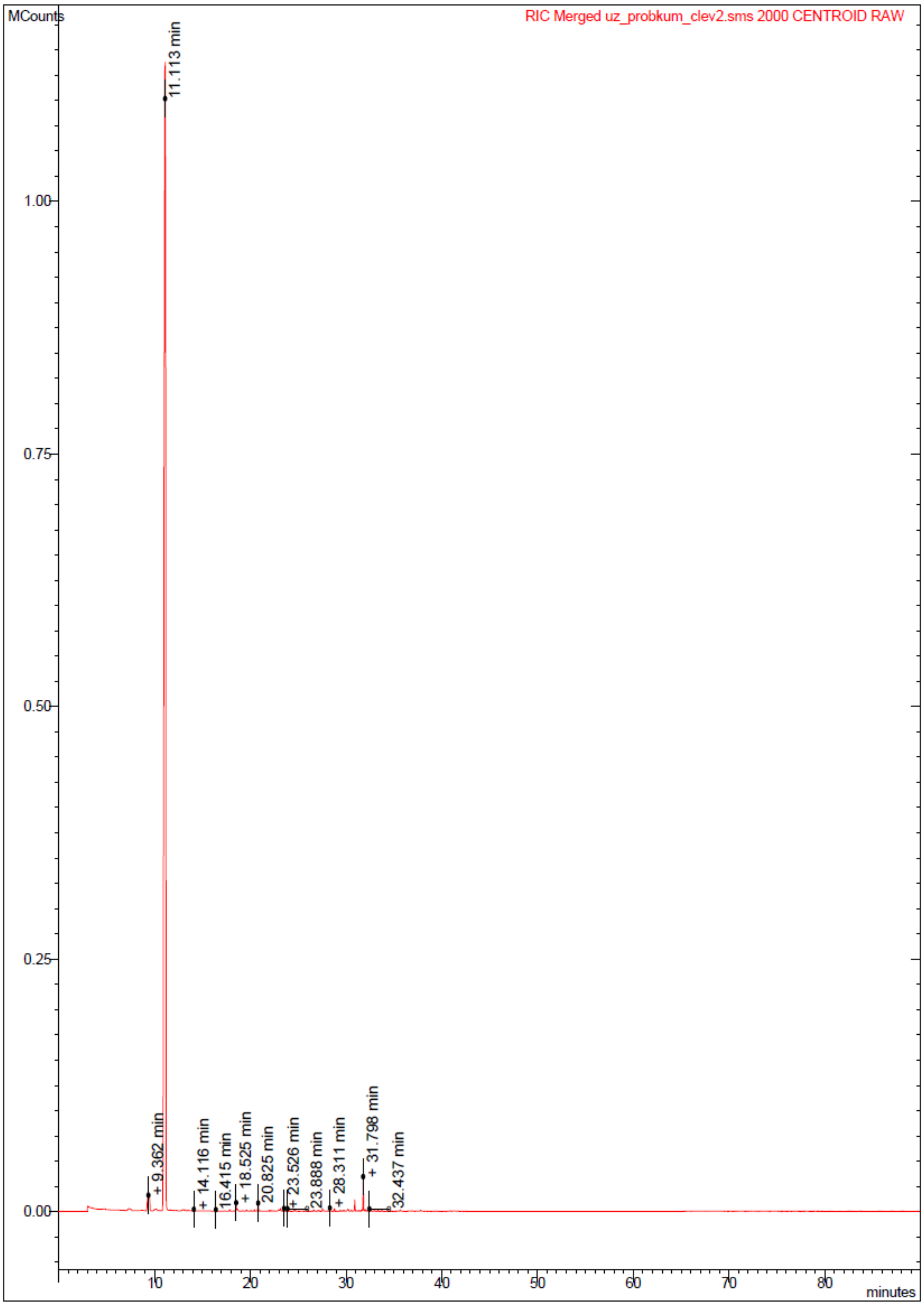

Figure S4. Chromatogram of chemical composition of EO $_{\text{Clev-HD}}$ after *in vitro* digestion.

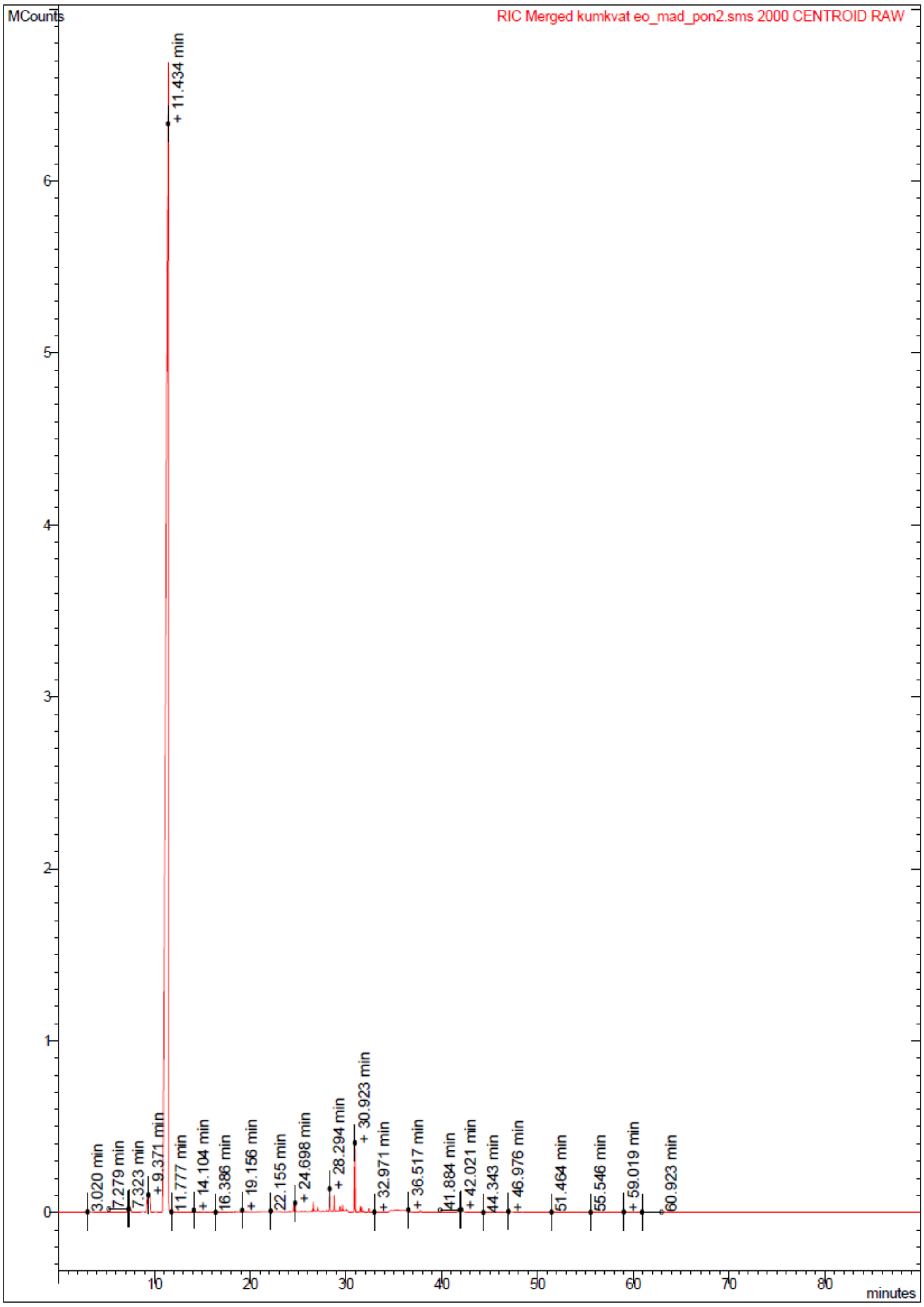

Figure S5. Chromatogram of chemical composition of EO MAD before *in vitro* digestion.

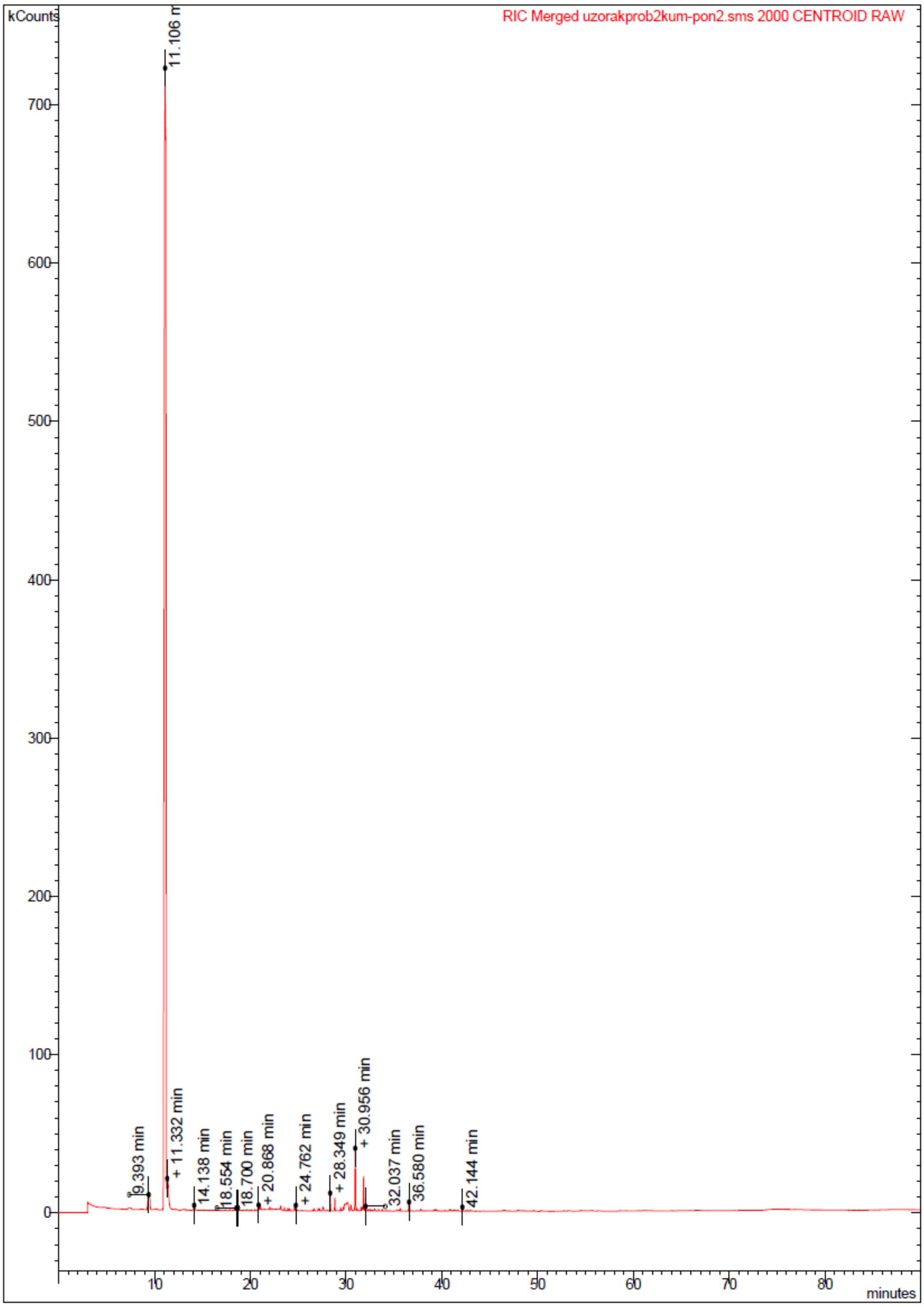

Figure S6. Chromatogram of chemical composition of EO MAD before *in vitro* digestion.
